# Downregulation of Notch Signaling‐Stimulated Genes in Neurovascular Unit Alterations Induced by Chronic Cerebral Hypoperfusion

**DOI:** 10.1002/iid3.70082

**Published:** 2024-11-28

**Authors:** Dewen Ru, Zengyu Zhang, Meng Liu, Xuhui Fan, Yuqi Wang, Yufeng Yan, Ersong Wang

**Affiliations:** ^1^ Department of Neurosurgery, Jinshan Hospital Fudan University Shanghai China; ^2^ Department of Neurosurgery, Huashan Hospital Fudan University Shanghai China; ^3^ Shanghai Medical College Fudan University Shanghai China; ^4^ Department of Neurology, Minhang Hospital Fudan University Shanghai China

**Keywords:** brain microvessel, chronic cerebral hypoperfusion, microglial, neuroinflammation, Notch signaling pathway

## Abstract

**Background:**

Chronic cerebral hypoperfusion (CCH) is a key contributor to vascular cognitive impairment (VCI) and is typically associated with blood–brain barrier (BBB) damage. This study investigates the pathological mechanisms underlying CCH‐induced neurovascular unit (NVU) alterations.

**Methods:**

A mouse model of CCH was established using the bilateral common carotid artery stenosis (BCAS) procedure. Decreased cerebral blood flow (CBF) and impaired BBB integrity were assessed. Brain microvessel (BMV)‐specific transcriptome profiles were analyzed using RNA‐seq, supplemented with published single‐cell RNA‐seq data.

**Results:**

RNA‐seq revealed neuroinflammation‐related gene activation and significant downregulation of Notch signaling pathway genes in BMVs post‐BCAS. Upregulated differentially expressed genes (DEGs) were enriched in microglia/macrophages, while downregulated DEGs were prominent in endothelial cells and pericytes. Enhanced activation of vascular‐associated microglia (VAM) was linked to neurovascular alterations.

**Conclusion:**

CCH induces significant NVU changes, marked by microglia‐associated neuroinflammation and Notch signaling downregulation. These insights highlight potential therapeutic targets for treating neuroinflammatory and vascular‐related neurodegenerative diseases.

## 1 Introduction

Chronic cerebral hypoperfusion (CCH) is increasingly recognized not only as a major contributor to vascular cognitive impairment (VCI) but also as a key initiator of immune and inflammatory responses within the neurovascular unit (NVU) [1]. The NVU, composed of endothelial cells, pericytes, astrocytes, microglia, and neurons, works collectively to regulate cerebral blood flow (CBF) and maintain the integrity of the blood‐brain barrier (BBB). The BBB serves as a key functional component of the NVU, restricting the passage of harmful substances between the bloodstream and the brain. This tight regulation ensures proper neural homeostasis and protects the brain from potential damage, with the NVU playing a crucial role in coordinating these interactions [2, 3]. When disrupted, particularly during CCH, the NVU undergoes pathological changes, leading to neuroinflammation and subsequent cognitive decline [4, 5]. Despite its importance, the precise molecular mechanisms underlying NVU damage and glial cell activation in the context of CCH remain insufficiently understood [6].

One critical aspect of CCH‐induced damage is microglial activation, which has been shown to play a pivotal role in driving inflammatory responses within the brain [7]. Activated microglia secrete proinflammatory cytokines and chemokines such as IL‐1β, TNF‐α, and IL‐6, which exacerbate neurovascular damage by promoting endothelial dysfunction and neuronal injury [8]. The inflammatory cascade is further amplified by oxidative stress, mitochondrial dysfunction, and endoplasmic reticulum (ER) stress, all of which are exacerbated by reduced CBF. The upregulation of matrix metalloproteinases (MMPs), particularly MMP‐9, also plays a significant role in the breakdown of the extracellular matrix, leading to BBB dysfunction and leukocyte infiltration into the brain parenchyma [9, 10]. This BBB disruption is a hallmark of neuroinflammation, contributing to the exacerbation of vascular and neuronal damage in CCH. Studies have shown that the presence of inflammatory biomarkers such as S100β, occludin, and claudin‐5 in cerebrospinal fluid and serum strongly correlates with cognitive decline in VCI patients [11]. Therefore, understanding the molecular and cellular mechanisms underlying BBB dysfunction in CCH is crucial for developing targeted therapeutic strategies to mitigate neurovascular damage and improve cognitive outcomes in patients with VCI.

Numerous rodent models have been developed to mimic the clinical features of CCH [12]. Among these, the bilateral common carotid artery occlusion (BCAO) and bilateral common carotid artery stenosis (BCAS) models are extensively used to simulate ischemic injury observed in clinical patients [13, 14]. However, the BCAO model has limitations due to the acute drop in CBF following artery ligation, which later resolves chronically. In contrast, the BCAS model, especially with 0.16/0.18 mm microcoils, has emerged as a more relevant rodent model for CCH [15], as it better replicates the asymmetric ischemia observed in clinical settings and is sufficient to induce gray matter damage [16, 17].

Recent advancements in transcriptomics, particularly RNA sequencing (RNA‐Seq), have provided powerful tools for genome‐wide transcript analysis [18]. RNA‐Seq enables the identification of differentially expressed genes (DEGs) and pathways associated with NVU alterations following cerebral hypoperfusion. By utilizing RNA‐Seq, researchers can gain deeper insights into the molecular and cellular responses of the NVU to chronic hypoperfusion, thereby uncovering potential therapeutic targets for mitigating neurovascular damage and cognitive decline in VCI.

In this study, we employed the BCAS model to investigate the molecular mechanisms of NVU damage and glial cell activation in CCH. Our objective was to elucidate the transcriptional changes and identify key pathways involved in the pathogenesis of CCH‐induced neurovascular alterations, providing a foundation for developing targeted therapeutic strategies for VCI.

## 2 Materials and Methods

### 2.1 Animal Experiments and Group Allocation

This study adhered to the ARRIVE guidelines for the use of animals in research [19]. Adult male C57BL/6 J mice, aged 9–10 weeks, were sourced from Beijing Vital River Laboratory Animal Technology. The mice were housed in individually ventilated cages (IVC) under specific pathogen‐free (SPF) conditions, with unrestricted access to food and water. Following a 1‐week acclimation period, the mice were randomly assigned to either the BCAS group or the Sham group. A total of 97 mice were initially included in the study. However, nine mice did not survive the BCAS procedure. The remaining 88 mice were allocated as follows for the various experimental procedures: 14 mice were used for CBF assessment and western blot analysis of brain tissues, 16 mice were designated for preparation of frozen sections, and 10 mice were used for paraffin section preparation and staining analysis. For the RNA‐seq, 24 mice were used, divided into three biological replicates, with each replicate consisting of pooled brain cortical microvessels from four mice. And 12 mice were allocated for western blot analysis of brain microvessels (BMVs), 12 mice were used for RT‐qPCR experiments on BMVs. The experimental protocol received ethical approval from the Shanghai Public Health Clinical Center's review board, with approval number 2023‐A040‐01. All procedures strictly adhered to the ethical guidelines established by the review board.

### 2.2 BCAS Procedure

The BCAS surgery was carried out with minor modifications based on previously described methods [20, 21]. Briefly, male mice were deeply anesthetized with 1.5% isoflurane in 30% oxygen. After exposing both common carotids from their sheaths, a microcoil with a 0.16 mm inner diameter (Anruike Biotechnology, Xi'an, China) was wound around the right common carotid arteries (CCA). Another 0.18 mm microcoil was wound around the left CCA 30 min later. Sham‐operated animals underwent identical surgical procedures without the placement of microcoils around the arteries. Both groups of surviving mice did not experience any intra‐operative or postsurgical complications. Following surgery, the mice were returned to their normal cages and allowed to recover with unrestricted access to food and water.

### 2.3 Measurement of CBF Using Laser Speckle Contrast Imaging

To evaluate the effects of hypoperfusion on CBF, laser speckle contrast imaging (LSCI) was employed 3 weeks post‐surgery (RFLSI III, RWD, China), following the protocol described previously [22]. Mice were anesthetized with isoflurane, and their heads were positioned in a stereotaxic frame in a prone position. A midline scalp incision was made to expose the skull. Then a whole‐brain scan was conducted using the LSCI system. Regions of interest were manually selected, and data analysis was performed using LSCI_V 1.0.0 software (RWD, China) to quantify changes in CBF. After completing the measurements, the scalp incision was carefully sutured. Throughout the procedure, the body temperature of the mice was maintained between 36.5°C and 37.5°C.

### 2.4 Hematoxylin‐Eosin (HE) Staining

Paraffin sections of brain tissues were prepared to conduct HE staining. Following perfusion, brains were extracted and fixed in freshly prepared 10% formalin for 24 h. Subsequently, the brain tissues were dehydrated and embedded in paraffin according to standard protocols. The paraffin‐embedded tissues were then sectioned into 4‐μm‐thick slices using a microtome. HE staining was carried out following established procedures [18]. Briefly, the 4‐μm‐thick sections were stained with hematoxylin solution for 5 min, followed by five immersions in 1% acidic ethanol (1% HCl in 70% ethanol), and then rinsed with distilled water. The sections were subsequently stained with eosin solution for 3 min, dehydrated through a series of graded alcohols, and cleared in xylene. Finally, the stained sections were mounted on slides and examined under a digital slide scanner (Pannoramic DESK, P‐MIDI, 3D HISTECH, Hungary) for photography and analysis.

### 2.5 Immunofluorescence Staining

Three weeks post‐surgery, mice were anesthetized with 2% isoflurane and then decapitated. Brains were extracted and fixed overnight in 4% formaldehyde in PBS at 4°C. Following fixation, the brains were dehydrated in 30% glucose at 4°C until they sank. Coronal sections, 50 µm thick, were prepared using a freezing microtome (CM 1900, Leica, Germany). For immunofluorescence staining, the free‐floating sections were first washed in PBS and then incubated for 1 h in a blocking buffer containing 1% Triton X‐100% and 5% normal goat serum (ThermoFisher, 16210064) in PBS. Subsequently, the sections were incubated overnight at 4°C with the following primary antibodies: rabbit anti‐IBA1 (Abcam, ab178846), rabbit anti‐Notch1 (CST, 4380S), and chicken anti‐GFAP (Abcam, ab4674). The next day, the sections were incubated for 1.5 h with secondary antibodies: Goat anti‐rabbit Alexa Fluor 594 (Jackson) and Goat anti‐chicken Alexa Fluor 647 (Jackson), followed by counterstaining with DAPI (ThermoFisher, 62248) for 20 min. Immunofluorescent images were acquired using an Olympus slide scanner (VS120‐L100). The images were processed with ImageJ software. For each animal, four brain slices were selected, and a whole‐brain scan was performed. Fields of view from the left and right hemispheres were analyzed.

### 2.6 Isolation of Brain Microvessels (BMVs)

BMVs were isolated from the right cerebral cortex following the protocol by Tu et al. [23]. Initially, large surface vessels were removed from the cortex, which was then homogenized using a dounce homogenizer in cold 17% dextran‐PBS solution (Sigma‐Aldrich) containing 2% fetal bovine serum (FBS). The homogenate was centrifuged at 8000 rcf for 15 min, after which the supernatant was discarded. The above pellet was resuspended in 1% BSA‐PBS and passed sequentially through 100‐μm and 45‐μm cell strainers (BD Falcon, CA). Microvessels were retained on the top of the 45‐μm strainer. The trapped microvessels were resuspended, centrifuged again, and collected for further assays.

### 2.7 RNA Extraction and Quantitative Real‐Time PCR (qRT‐PCR)

Total RNA was extracted from isolated BMV samples using Trizol Reagent (Invitrogen, USA). To eliminate any potential genomic DNA contamination, the samples were treated with an RNase‐free DNase set (QIAGEN). RNA concentration and purity were assessed using a NanoDrop 2000 spectrophotometer (Thermo Fisher Scientific). The RNA samples were stored at −80°C until further use. Reverse transcription was performed using the HiScript III All‐in‐one RT SuperMix Perfect for qPCR (Vazyme, R333). Quantitative real‐time PCR (qRT‐PCR) was conducted on a Thermo Fisher Scientific Applied Biosystems QuantStudio 5 system, employing the ChamQ Universal SYBR qPCR Master Mix (Vazyme, Q711). Primer sequences used for qRT‐PCR are listed in Table S1. Relative gene expression analyses were performed using the 2^‐ΔΔCt^ method, with *Gapdh* serving as the reference gene for normalization.

### 2.8 RNA‐Seq and Data Analysis

For RNA‐seq experiments, three samples were selected from each group. The cDNA library was constructed using the NEBNext Ultra RNA Library Prep Kit for Illumina (NEB, USA) according to the manufacturer's instructions and sequenced on an Illumina HiSeq platform with a PE150 pattern. Raw sequencing data were obtained in FASTQ format and quality control was performed using FastQC. Data preprocessing was conducted with Trim Galore to produce high‐quality clean reads by removing adapter sequences, low‐quality reads, and excessively short reads. Subsequent analyses were based on these clean reads. Sequencing reads were aligned to annotated RefSeq genes in the mouse reference genome (UCSC mm10) using HISAT2. SAMtools (v1.9) was employed to convert, sort, and index the alignments. FeatureCounts was used to count the number of reads mapped to each gene, and the FPKM of each gene was calculated based on gene length and read count. Differential expression analysis between the BCAS and sham groups was conducted using DESeq. 2. DEGs were identified with an adjusted *p*‐value < 0.05 and |Log2fold change | > 1. Gene Ontology (GO) enrichment analysis with DEGs was conducted using ShinyGO 0.76.3, considering GO terms with an adjusted *p*‐value < 0.05 as significantly enriched. The sequencing data have been deposited under the accession code GSE266214.

### 2.9 Integrative Analysis of Bulk RNA‐Seq and Published Single‐Cell RNA‐Seq (scRNA‐Seq) Data

Dimensionality reduction and cell clustering were performed using the UMAP algorithm, as previously described [24]. Cell clusters were generated with Seurat on a UMAP plot using published scRNA‐seq data (GSE133283) [25]. We conducted cell‐type enrichment analysis by integrating DEGs from our BMV‐specific bulk RNA‐seq data with the published scRNA‐seq data. The DEGs were categorized into two lists: “BCAS‐induced upregulated genes” (up_genes) and “BCAS‐induced downregulated genes” (down_genes). Then, cell‐type deconvolution analysis was performed using the *UCell* algorithm, available from the open‐source R package at https://github.com/chuiqin/irGSEA.

### 2.10 Western Blot Analysis

Brain tissues and microvessels were lysed using RIPA lysis buffer supplemented with a protease/phosphatase inhibitor cocktail (Beyotime‐P1005). Protein concentrations were determined using the BCA assay. Equal amounts of protein were then denatured with 5× loading buffer at 95°C for 10 min. The denatured proteins were subjected to SDS‐polyacrylamide gel electrophoresis and subsequently transferred onto PVDF membranes (Millipore, USA). The membranes were blocked with 5% BSA in TBS containing 0.1% Tween20 (TBST) and incubated overnight at 4°C with primary antibodies diluted in 1% BSA‐TBST. The primary antibodies used included anti‐CD31 (R&D Systems, AF3628) and anti‐GFAP (Abcam, ab68428). Following primary antibody incubation, the membranes were incubated with horseradish peroxidase‐conjugated secondary antibody (Goat anti‐rabbit, Beyotime‐A0208) for 1.5 h at room temperature. Protein signals were detected using Clarity ECL substrate (Bio‐Rad 170‐5060) and visualized. The resulting images were processed and analyzed using ImageJ software.

### 2.11 Statistical Analysis

All experimental procedures and data analyses were performed under blinded conditions to ensure objectivity. Statistical analyses were conducted using Prism 9 software for Windows (GraphPad Software). Normality tests were conducted for all datasets using the Shapiro–Wilk test. For comparisons between the 0.18 and 0.16 mm sides within the BCAS group, a paired *t*‐test (two‐tailed) was applied. Unpaired *t*‐tests (two‐tailed) were used for comparisons between the sham and BCAS groups. When comparing more than two groups, one‐way ANOVA was employed to assess intergroup differences. Data were presented as mean ± SEM, and significance was determined at *p* < 0.05, with *p < 0.05, **p < 0.01, and ***p < 0.001 indicating statistical significance.

## 3 Results

### 3.1 BCAS‐Induced Hypoperfusion Results in CBF and Histological Changes

**Figure 1:**
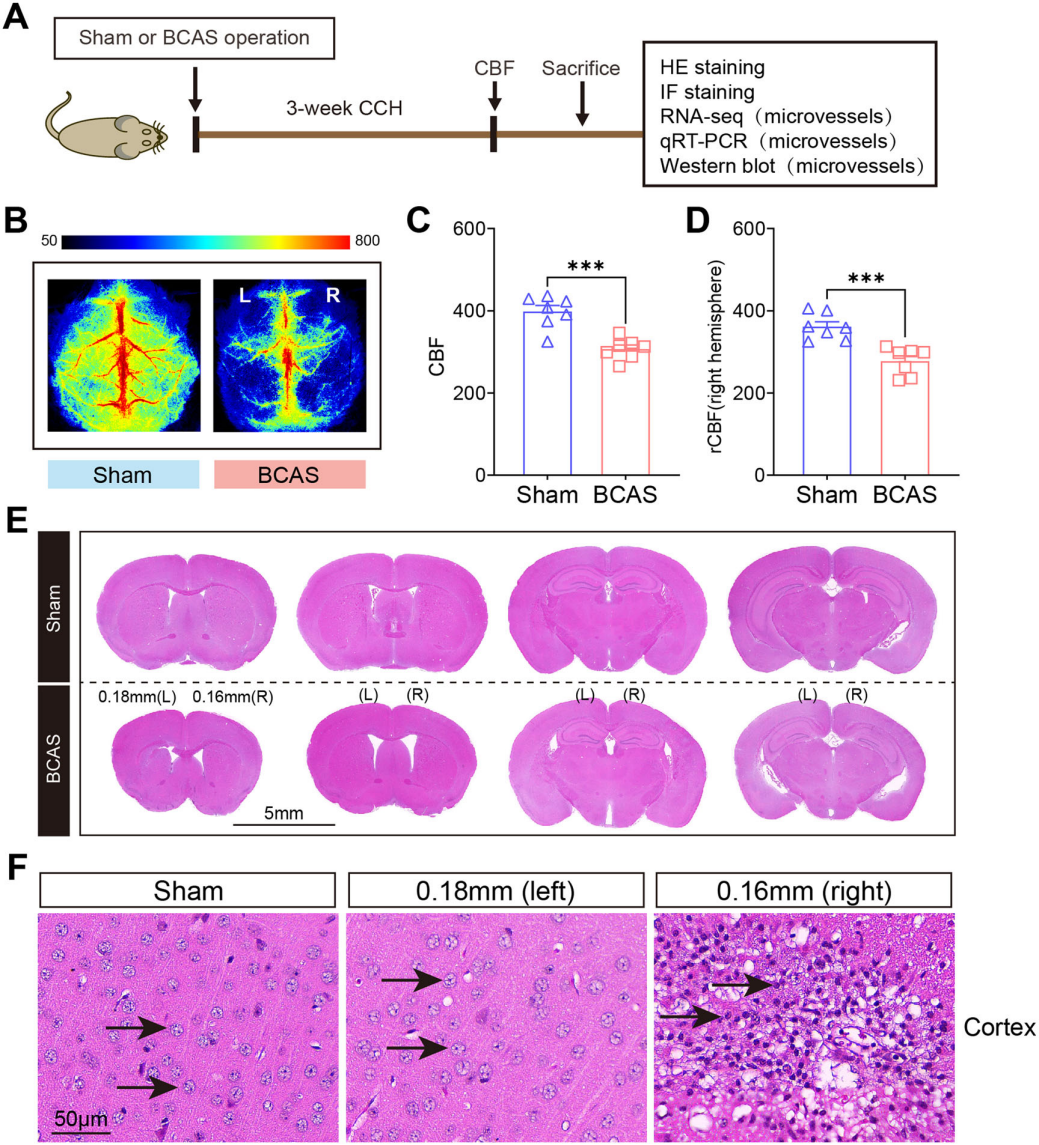
Effects of chronic cerebral hypoperfusion on cerebral blood flow and histopathology. (A) The schematic diagram of the experimental protocol. BCAS, bilateral carotid artery stenosis. CBF, cerebral blood flow. CCH, chronic cerebral hypoperfusion. HE staining, hematoxylin and eosin staining. IF, Immunofluorescence staining. (B) Representative images showing CBF at 3 weeks post‐BCAS or sham surgery. (C) Quantitative analysis of CBF across the whole brain in both sham and BCAS groups. (D) Quantitative analysis of CBF in the right hemisphere in sham and BCAS groups. N = 7 mice per group. (E) Coronal brain sections stained with HE to reveal brain injury induced by BCAS. (F) Representative HE‐stained images of the cerebral cortex under high magnification. Data are expressed as mean ± SEM. Statistical significance was determined using an unpaired t‐test (two‐tailed). ***p < 0.001 compared to the sham group.

The experimental design and procedures are outlined in Figure 1A. Initially, CBF changes were assessed in BCAS mice. Our findings indicated a notable reduction in CBF on the right side of the brain 3 weeks post‐BCAS hypoperfusion (Figure 1B–D), aligning with previous research. Specifically, BCAS mice treated with 0.16/0.18 mm microcoils exhibited an approximate 23.2% decrease on average CBF compared to the right hemisphere of sham‐operated mice. To investigate histopathological changes following cerebral hypoperfusion, HE staining was conducted on four different brain sections (Figure 1E). Observations revealed cortical atrophy in the right hemisphere. Additionally, neurons in the infarct region of BCAS mice were markedly damaged, disorganized, and significantly reduced in number, displaying pyknotic and indistinct nuclei indicative of neuronal death (Figure 1F). In summary, BCAS‐induced hypoperfusion resulted in pronounced neuropathological alterations compared to the sham‐operated controls. The results of our study validate the successful establishment of a mouse model for cerebral hypoperfusion.

### 3.2 Astrocyte Activation and BBB Disruption Caused by BCAS‐Hypoperfusion

**Figure 2:**
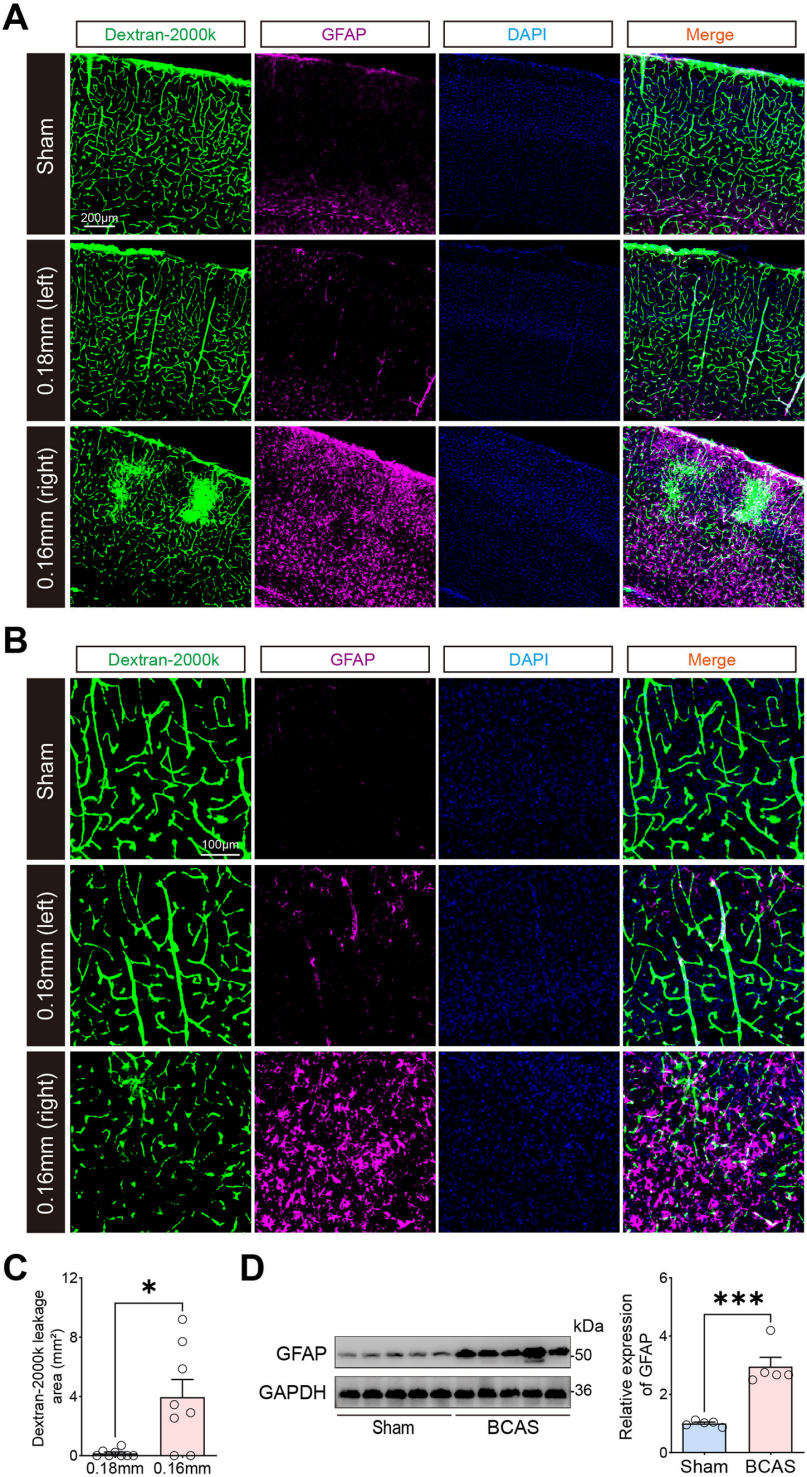
Activated astrocyte and the disruption of blood‐brain barrier integrity induced by BCAS‐hypoperfusion. (A) Representative double‐labeling staining images of GFAP and FITC‐dextran in brain sections at 3 weeks post‐BCAS. (B) Microscopic images of perfused cortical capillaries with intravenously injected FITC‐dextran and immunofluorescence staining of GFAP in the cortical region. (C) Assessment of blood‐brain barrier permeability by FITC‐dextran leakage. Data are expressed as mean ± SEM. Paired *t‐*test (two‐tailed), *p < 0.05 versus 0.18 mm side, *n* = 8 per group. (D) Representative western blot images of GFAP in the cerebral cortex are shown on the left, and the statistical analysis of relative GFAP expression level is on the right. Unpaired *t*‐test. N = 5 per group. Data are expressed as mean ± SEM. Unpaired *t‐*test (two‐tailed), ***p < 0.001 versus sham, *n* = 5 mice per group.

To assess the integrity of the BBB, perfusion of cortical capillaries with intravenously injected FITC‐dextran (2000kDa) was conducted, followed by an analysis of cerebrovascular lesions resulting from BCAS‐induced hypoperfusion. The findings revealed significant leakage of the fluorescent tracer near the stenotic region of the 0.16 mm side in BCAS mice, with substantial morphological degradation and reduced vascular density (Figure 2A,B). Immunofluorescence staining using the astrocyte marker GFAP identified injury areas characterized by reactive astrocytes. Notably, the 0.16 mm stenosis induced evident astrocyte activation, predominantly surrounding the FITC‐dextran leakage areas, including the cerebral cortex, forming typical astrocytic scars. Comparative analysis of the fluorescent tracer leakage between the 0.18 and 0.16 mm sides in the BCAS group showed more pronounced FITC‐dextran leakage on the 0.16 mm side (Figure 2C). Furthermore, western blot analysis indicated a significant increase in GFAP protein expression in the cerebral cortex of BCAS mice (Figure 2D). These results collectively suggest that BCAS‐hypoperfusion with 0.16/0.18 mm microcoils leads to significant BBB integrity disruption. Our results are consistent with prior observations, and provide a comprehensive basis for studying neuropathological changes in the brain microvessels following cerebral hypoperfusion.

### 3.3 Transcriptional Activation of Inflammation‐ and Immune Response‐Stimulated Genes in BMVs After Cerebral Hypoperfusion

**Figure 3:**
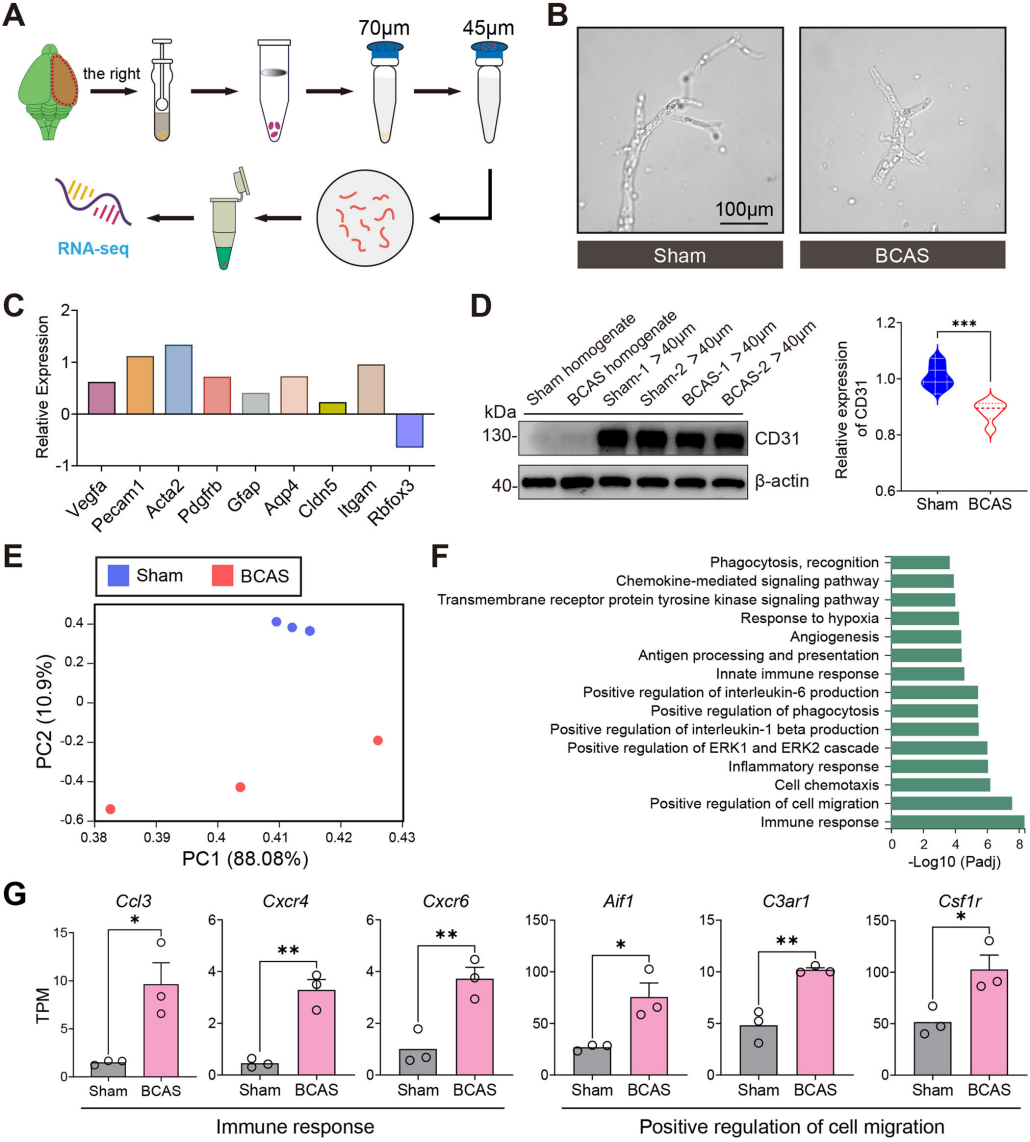
Successful isolation of BMV from the right cerebral cortex and BMV‐specific transcriptome profiling 3 weeks post BCAS‐hypoperfusion. (A) Schematic diagram of BMVs isolation. BMV, brain microvessels. (B) Representative light microscopy images show freshly isolated BMVs from the cerebral cortex of both sham‐operated and BCAS‐treated mice. (C) RT‐qPCR analysis revealed the relative transcriptional expression levels of various genes in brain homogenate (Homogenate) compared to microvascular separation products (Microvessel). The relative expression was calculated using the formula: Relative Expression = Microvessel/Homogenate. (D) Western blot analysis presented representative images of CD31, an endothelial marker, in the cerebral cortex, with the statistical analysis of relative CD31 expression levels showing significant differences. 40 μm means the specific size threshold used to filter microvessel fractions. N = 6 per group. Unpaired *t*‐test (two‐tailed), ***p < 0.001 versus sham. (E) Principal component analysis (PCA) of RNA‐seq datasets from brain cortical microvessels highlighted distinct clustering between sham and BCAS groups. (F) Gene ontology (GO) analysis provided functional annotations of all differentially expressed genes between BCAS and sham groups (BCAS vs. Sham). (G) Bar charts depicted the gene expression profiles in Transcripts Per Million (TPM), noting significant increases in immune response and cell migration‐related genes (*Ccl3*, *Cxcr4*, *Cxcr4*, *Aif1*, *C3ar1*, *Csf1r*) in the BCAS group compared to the sham group. Data are expressed as mean ± SEM, with significance determined by unpaired *t*‐test (two‐tailed), *p < 0.05, **p < 0.01 versus sham, *n* = 3 per group.

While prior studies have shown impairment in BMVs function post‐cerebral ischemia, the underlying molecular mechanisms remain largely elusive. In our study, we established a chronic cerebral ischemia model and examined the transcriptomic alterations in BMVs to understand their impact on BBB permeability. Cerebral cortex from BCAS‐treated and sham‐operated mice was collected, and BMVs were isolated (Figure 3A,B). RT‐qPCR analysis demonstrated that, compared to brain homogenate, the isolated BMVs exhibited higher transcriptional expression of cellular markers linked to NVU, including *Acta2* (vascular smooth muscle cells), *Pdgfrb* (pericytes), *Vegfa* and *Pecam1* (endothelial cells), *Gfap* and *Aqp4* (astrocytic end‐feet), and *Itgam* (microglia) (Figure 3C). Western blot further confirmed the purity of the isolated BMVs and indicated a reduction in microvascular endothelial cells following BCAS‐induced hypoperfusion. We then focused on BMV‐specific transcriptional changes induced by 0.16/0.18 mm BCAS surgery. RNA‐Seq analysis was conducted using three biological replicates at 3 weeks postprocedure, each consisting of pooled brain right cortical microvessels from four mice. Principal component analysis (PCA) yielded scores of 88.08% for PC1% and 10.9% for PC2, indicating a distinct transcriptome signature in the BMVs after BCAS‐induced hypoperfusion (Figure 3E). Significant transcriptional changes were observed in the BCAS group compared to the sham group. DEGs were identified using thresholds of |log2FC | > 1 and adjusted P‐value (FDR) < 0.05, resulting in a total of 480 DEGs (Table S2). GO analysis of these DEGs revealed significant enrichment in immune response, phagocytosis, and angiogenesis (Figure 3F). Verification via RNA‐seq data showed that genes related to immune response and cell migration (*Ccl3*, *Cxcr4*, *Aif1*, *C3ar1*, *Csf1r*) were significantly upregulated in the BCAS group compared to the sham group, suggesting the involvement of microglia in NVU (Figure 3G). These results elucidate the molecular mechanisms underlying BMVs dysfunction following cerebral hypoperfusion and highlight the significant role of inflammation and immune response in this process.

### 3.4 BMV‐Specific Transcriptome Profiling Reveals Downregulation of Notch Signaling‐Related Genes Following BCAS‐Hypoperfusion

**Figure 4:**
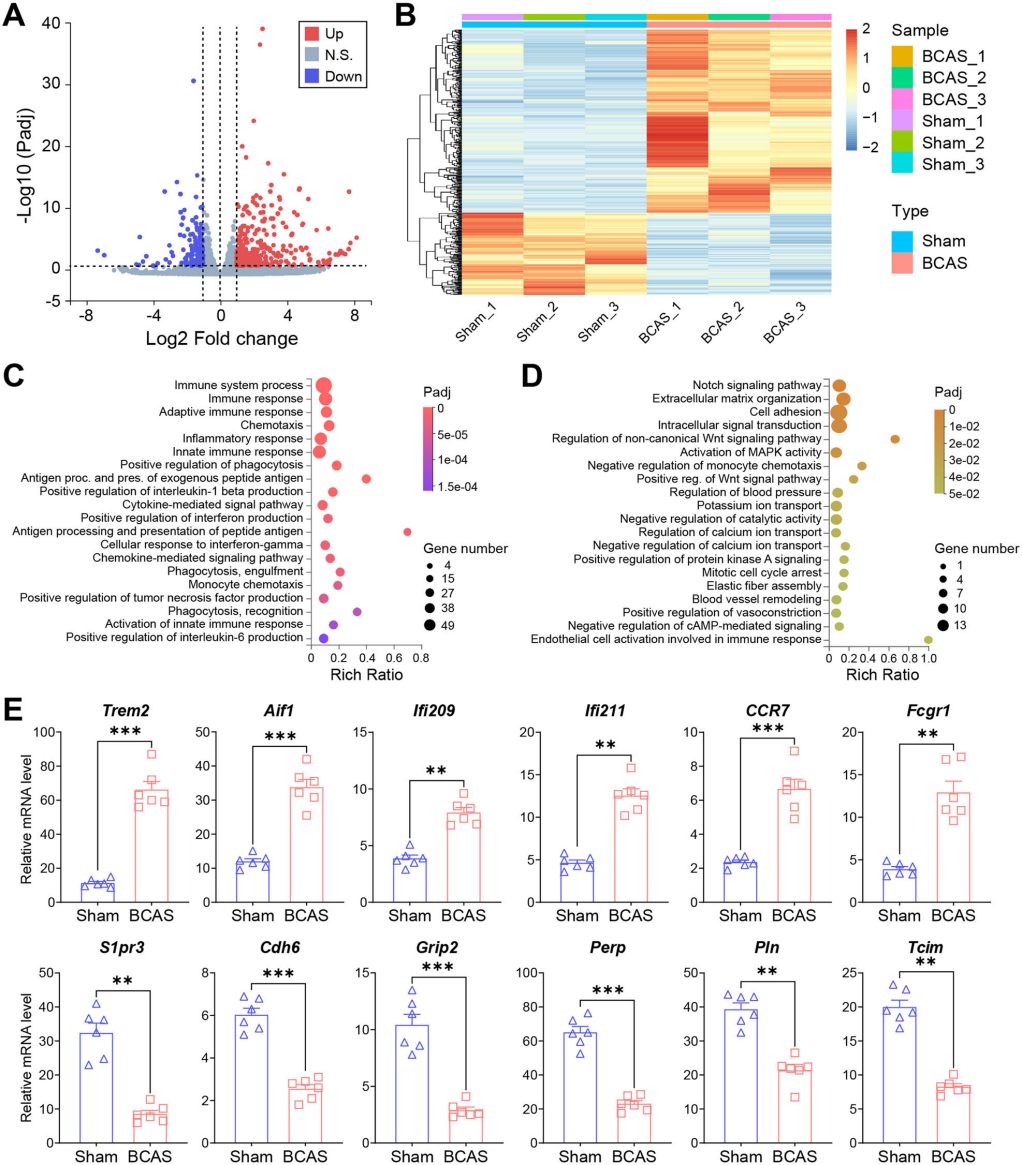
Gene expression changes and gene ontology (GO) enrichment analysis of up‐ and downregulated DEGs after BCAS‐hypoperfusion 3 weeks. (A) Volcano plot illustrating the differentially expressed genes (DEGs) in brain microvessels (BMVs) between the BCAS and sham groups. The log2FC cutoff is set at 1, and the adjusted *P*‐value cutoff is 0.05. Each dot represents an expressed gene, with upregulated genes highlighted in red and downregulated genes in blue. (B) Heatmap displaying the correlation between samples, demonstrating the relationship and clustering of gene expression profiles among the different samples. (C) Bar plot showing the enriched Gene Ontology (GO) biological process terms for upregulated genes, indicating significant involvement in processes such as immune response and cell migration. (D) Bar plot depicting the enriched GO biological process terms for downregulated genes, highlighting significant pathways such as the Notch signaling pathway. (E) Validation of several upregulated and downregulated genes through quantitative real‐time PCR (gene expression was normalized by the housekeeping gene *Gapdh*). The upregulated genes are associated with “Positive regulation of phagocytosis” and “Interferon response” (*Trem2*, *Aif1*, *Ifi209*, *Ifi211*, *Ccr7*, *Fcgr1*), while the downregulated genes are involved in the “Notch signaling pathway” (*S1pr3*, *Cdh6*, *Grip2*, *Perp*, *Pln*, *Tcim*). Data are presented as mean ± SEM, with significance determined by unpaired *t*‐test (two‐tailed, **p < 0.01, ***p < 0.001), N = 6 mice per group.

**Figure 5:**
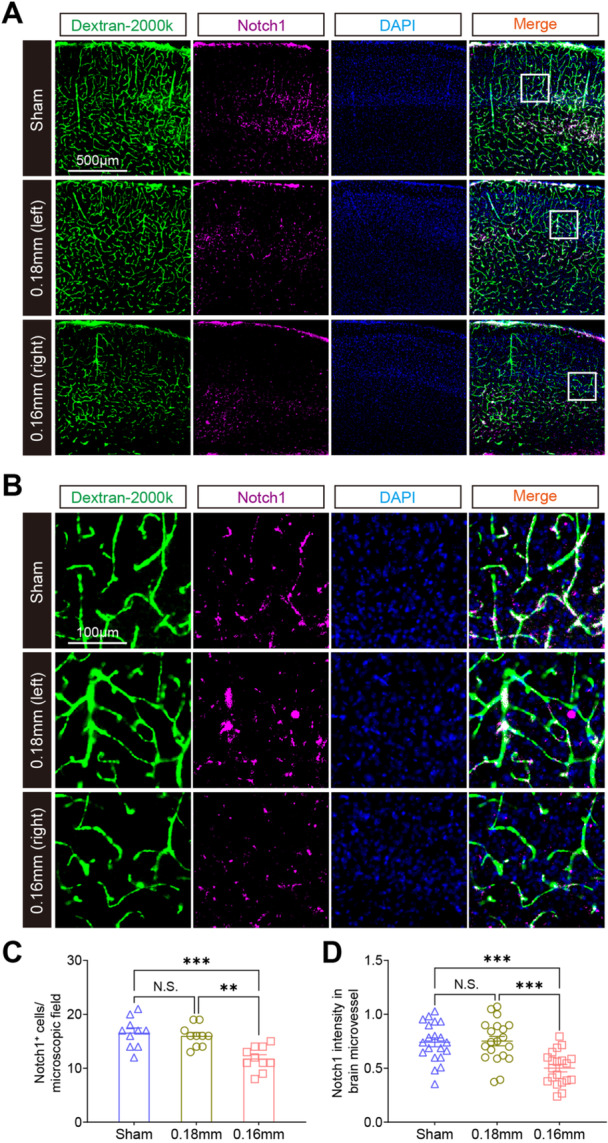
Notch signaling mediates the decrease of gene expression in BCAS‐induced hypoperfusion. (A) Representative immunofluorescent staining images of Notch1 in brain sections 3 weeks post sham and BCAS operation, with 2000‐kDa FITC‐dextran (green) injection. (B) High‐resolution microscopic images showing the co‐localization of FITC‐dextran and Notch1 in the cortical region. (C) Quantification of Notch1‐positive cells per field. (D) Quantitative analysis of Notch1 intensity in selected brain microvessels. Data are expressed as mean ± SEM. Statistical significance was determined using a one‐way ANOVA test, with NS indicating not significant, **p < 0.01, and ***p < 0.001.

In this study, significant alterations in gene expression were analyzed as previously described. A total of 480 DEGs were identified in BMVs between the BCAS and sham groups, with 332 (69.2%) being significantly upregulated and 148 (30.8%) significantly downregulated (Figure 4A; Table S2). The clustering heatmap showed high similarity among samples within the BCAS group while demonstrating clear separation from the sham group samples (Figure 4B). The majority of DEGs were upregulated following BCAS‐hypoperfusion. GO enrichment analysis was then conducted on these DEGs, identifying the top 20 biological processes (BP) GO terms for both upregulated and downregulated genes (Figure 4C,D). Our data revealed significant enrichment of immune‐related biological processes among the upregulated DEGs, including “Positive regulation of phagocytosis,” “Positive regulation of interleukin‐1 beta production,” “Cytokine‐mediated signaling pathway,” and “Positive regulation of interferon production” (Figure 4C). Conversely, crucial pathways associated with endothelial angiogenic activity were downregulated after BCAS‐hypoperfusion, with the most significant being the “Notch signaling pathway,” “Cell adhesion,” and “Extracellular matrix organization,” indicating impaired vascular function (Figure 4D). To further validate these findings, we performed quantitative real‐time PCR on isolated BMVs to assess the mRNA expression levels in BCAS mice compared to sham‐operated mice. The genes analyzed included *Trem2*, *Aif1*, *Ifi209*, *Ifi211*, *Ccr7*, *Fcgr1*, *S1pr3*, *Cdh6*, *Grip2*, *Perp*, *Pln*, *Tcim* (Figure 4E). Further, recent studies have highlighted the role of Notch signaling in brain endothelial cells, particularly in the context of vascular and neuroinflammatory responses. Our results indicated that Notch1 signaling mediates transcriptional repression of gene expression in BMVs following BCAS‐induced hypoperfusion. Then, immunofluorescence staining showed that Notch1 was significantly downregulated in FITC‐dextran labeled brain sections post chronic cerebral ischemia (Figure 5). In summary, our results consistently demonstrated that chronic hypoperfusion led to transcriptional activation of genes involved in inflammatory and immune responses, while concurrently repressing genes related to Notch signaling and endothelial cell migration. This dual regulatory effect underscores the complex molecular changes induced by BCAS‐hypoperfusion, highlighting the significant impact on both immune response and vascular function.

### 3.5 BMV‐Specific DEGs Induced by BCAS Hypoperfusion Are Enriched in Distinct Brain Cell Types

Previous studies identified different cell types in the mouse brain using scRNA‐seq under normal or pathological conditions [26, 27]. Our integrative analysis combined bulk RNA‐seq data from the current study with previously published scRNA‐seq data [25], revealing the enrichment of BMV‐specific DEGs in distinct brain cell types.

**Figure 6:**
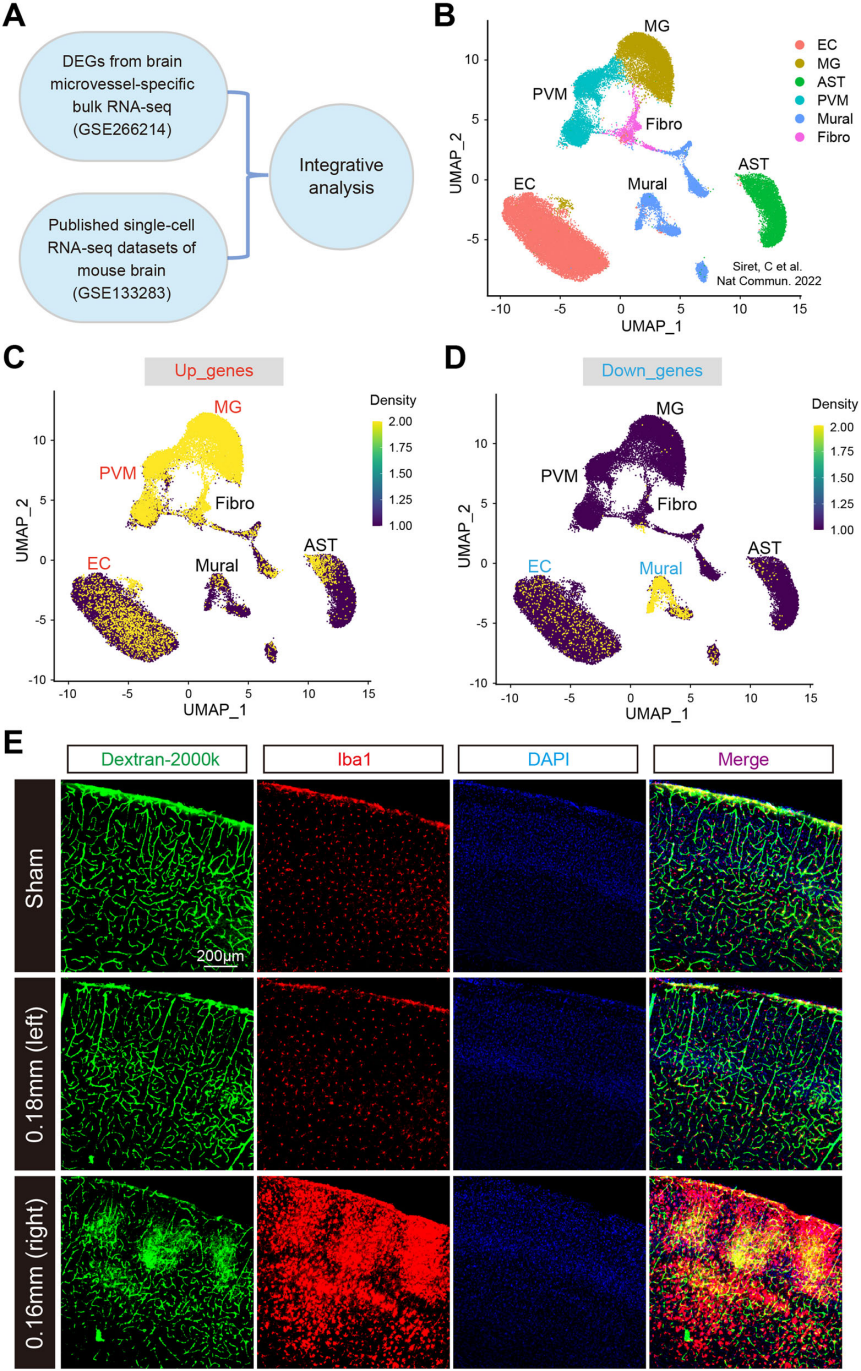
Enrichment of cerebral hypoperfusion‐induced BMV‐specific DEGs in distinct brain cell types. (A) Schematic representation of the integrative analysis combining bulk RNA‐seq data from the current study with previously published single‐cell RNA‐seq data. (B) UMAP plot illustrating the clustering of single cells, colored by cell type, based on published single‐cell RNA‐seq data (GSE133283). (C‐D) Density scatterplots displaying cell‐type enrichment for (C) upregulated genes (“Up_genes”) and (D) downregulated genes (“Down_genes”) using the *UCell* R package (https://github.com/chuiqin/irGSEA). The genes visualized are DEGs identified from BMV‐specific bulk RNA‐seq in this study. Note the enrichment of upregulated genes in the “MG” and “PVM” clusters, and downregulated genes in the “Mural” and “EC” clusters. PVM, perivascular macrophage. MG, microglia. EC, endothelial cell. Fibro, fibroblasts. Mural, brain pericyte. AST, astrocyte. (E) Immunofluorescence Iba1 antibody staining (microglial marker) was performed on FITC‐Dextran‐labeled brain slices. The cell types enriched by upregulated DEGs were verified.

Using scRNA‐seq data from non‐neuronal cell populations in the mouse brain cortex, we identified six transcriptionally distinct clusters based on known cell type markers (Figure 6A,B). These clusters included perivascular macrophages (PVM), microglia (MG), endothelial cells (EC), fibroblasts (Fibro), brain pericytes (Mural), and astrocytes (AST). In addition, cell‐type enrichment analysis using the *UCell* algorithm in irGSEA showed a distinct distribution pattern of “BCAS‐induced upregulated genes” predominantly in the MG and PVM clusters (Figure 6C). Conversely, “BCAS‐induced downregulated genes” were significantly enriched in Mural cells, followed by EC clusters (Figure 6D). These findings indicate that different cell types within the BMVs exhibit differential transcriptional responses to BCAS‐hypoperfusion. Specifically, transcriptional activation is primarily observed in microglia, while transcriptional repression occurs in brain endothelial cells and pericytes.

### 3.6 Alterations in the Neurovascular Unit Induced by BCAS‐Hypoperfusion Are Associated With Enhanced Activation of Vascular Associated Microglia (VAM)

**Figure 7:**
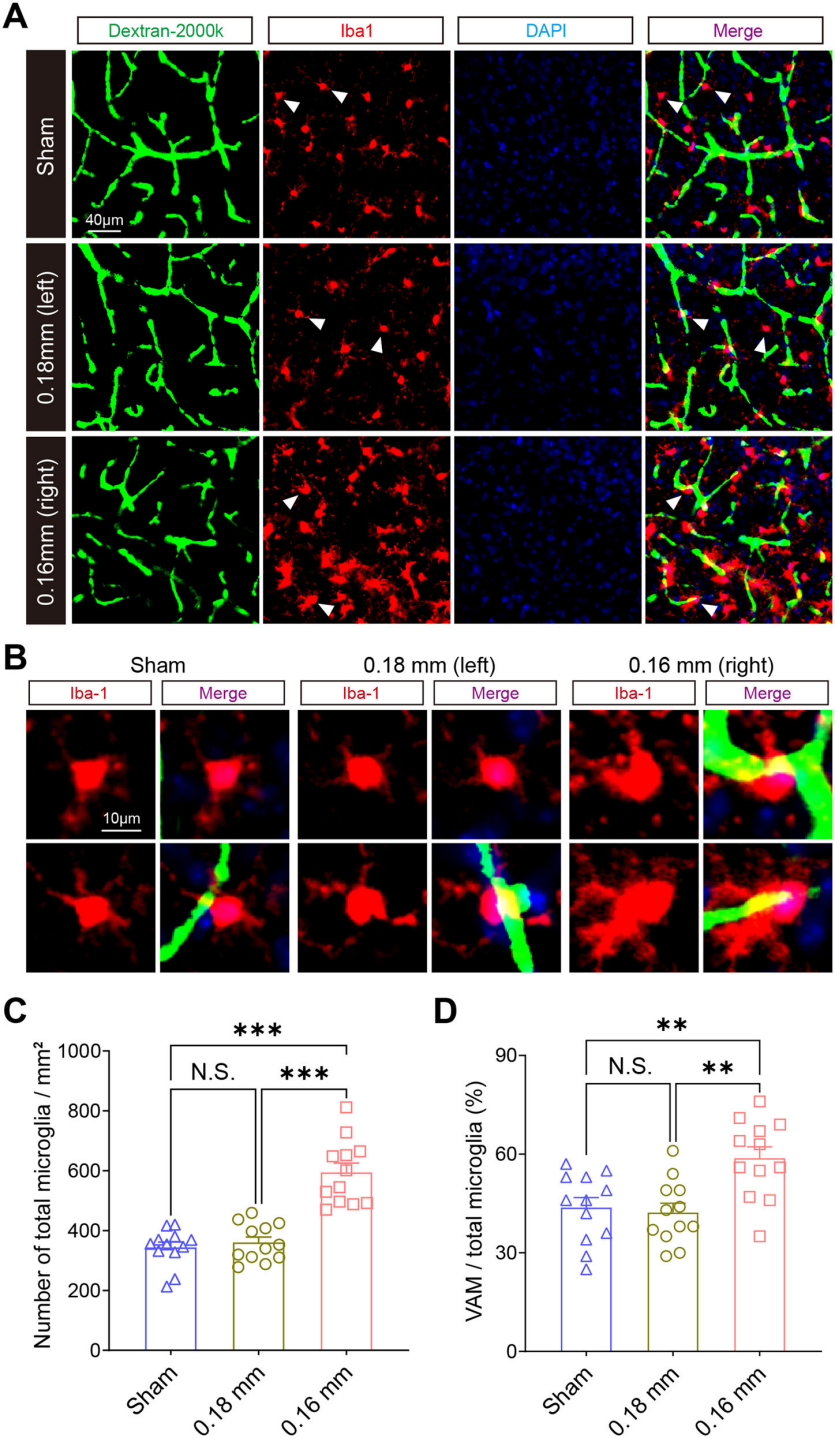
BCAS‐hypoperfusion leads to significant activation of vascular‐associated microglia 3 weeks post‐BCAS. (A,B) Representative images illustrating blood‐brain barrier (BBB) disruption and vessel‐associated microglia (VAM) location in the cerebral cortex at three weeks post BCAS‐hypoperfusion. The arrowheads indicate microglia located adjacent to vessels, highlighting their potential interactions with the vascular structures. (C) Quantification of the number of Iba1‐positive microglia per field. (D) T Percentage of VAM among total Iba1‐positive microglia in each microscope field of view. Data are presented as mean ± SEM. Statistical significance was determined using a one‐way ANOVA test, with NS indicating not significant, **p < 0.01, ***p < 0.001.

Our findings suggest that gene expression changes induced by BCAS‐hypoperfusion are closely linked to the activation of microglia, with hypoperfusion‐induced upregulated genes significantly enriched in immune‐related biological processes. Prolonged cerebral hypoperfusion resulted in a noticeable increase in perivascular macrophages and a significant reduction in endothelial cells within NVU. This cellular shift explains the downregulation of genes associated with angiogenesis and cell migration. Neuroinflammation is known to profoundly impact BBB integrity, affecting both its damage and repair. To further confirm our findings, BMVs were visualized in brain sections using 2000kDa FITC‐dextran injection. The staining revealed Iba1‐positive microglia adhering to cerebrovascular structures, identifying these cells as vascular‐associated microglia (VAM) (Figure 7A,B). Notably, the stenotic side (0.16 mm) of BCAS‐treated mice showed a significant increase in the total number of Iba1‐positive microglia and a higher percentage of VAM (Figure 7C,D). In conclusion, our results demonstrate that deeper cerebral hypoperfusion causes changes in the cellular composition of NVU, particularly marked by an enrichment of perivascular immune cells.

## 4 Discussion

CCH is a significant contributor to VCI, with a complex underlying pathology involving the NVU and the BBB. This study delves into the mechanisms driving these alterations, particularly focusing on neuroinflammation and Notch signaling. Our results demonstrate that BCAS‐induced hypoperfusion leads to significant NVU alterations. These changes are characterized by microglia‐associated neuroinflammation and downregulation of Notch signaling, providing new insights into the molecular mechanisms underlying neurovascular dysfunction in chronic hypoperfusion. These findings highlight potential therapeutic targets for mitigating neuroinflammatory and vascular‐related neurodegenerative diseases.

The integrity of the BBB is vital for maintaining central nervous system homeostasis. In CCH, hypoperfusion‐induced BBB disruption is often accompanied by endothelial dysfunction and the activation of inflammatory cells, particularly microglia [28, 29]. Our findings demonstrated a significant reduction in CBF following the 0.16/0.18 mm BCAS model, which correlated with compromised BBB integrity. This disruption facilitates the infiltration of peripheral immune cells, exacerbating neuroinflammation and contributing to the progression of neurovascular damage [30].

RNA‐sequencing (RNA‐seq) analysis of brain microvessels (BMVs) post‐BCAS revealed significant transcriptional activation of genes associated with neuroinflammation and immune responses. This transcriptional activation underscores the critical role of microglia in mediating inflammatory responses under hypoperfusion conditions [31]. Genes such as those involved in cytokine signaling and immune cell recruitment were markedly upregulated, highlighting the heightened state of neuroinflammation [32].

Interestingly, our study demonstrates significant downregulation of genes associated with the Notch signaling pathway in BMVs following BCAS‐induced hypoperfusion. Notch signaling is crucial for cell differentiation and maintaining vascular homeostasis [33]. Previous research has highlighted the roles of Notch4 and Jagged‐1 in endothelial differentiation, showing that they can induce the formation of microvessel‐like structures with properties of brain endothelial microvessels. Activation of this pathway promotes endothelial cell differentiation and morphogenesis, underscoring its importance in vascular development [34]. Furthermore, studies on mesenchymal stem cells (MSCs) have revealed their role in mitigating early brain injury following subarachnoid hemorrhage (SAH) by suppressing Notch1‐dependent neuroinflammation. MSC treatment has been shown to reduce neurobehavioral deficits, brain water content, and BBB disruption through the inhibition of Notch1 signaling and upregulation of Botch, an endogenous Notch1 inhibitor, leading to decreased microglial activation and pro‐inflammatory cytokine production [35].

In our study, we observed a significant downregulation of Notch1 in endothelial cells, which aligns with its known role in vascular homeostasis and injury response. Previous research, including our own, has shown that activating Notch1 can enhance endothelial regeneration and promote angiogenesis, contributing to the restoration of BBB integrity [23]. Additionally, Notch3, primarily expressed in vascular smooth muscle cells (VSMCs) and pericytes (mural cells), plays a critical role in maintaining vessel structure and function [36]. Suppression of Notch3 compromises mural cell support, leading to vascular instability. Our findings revealed that downregulated genes in our model, such as *S1pr3*, *Cdh6*, *Grip2*, *Perp*, *Pln*, and *Tcim*, overlap with genes reported in Notch3 knockout mice, supporting the importance of Notch3 in vascular integrity [37]. These observations are consistent with previous studies highlighting the role of Notch3 mutations in conditions like CADASIL, where VSMC dysfunction leads to BBB compromise and brain pathology [38]. Our study reinforces the idea that Notch3 is vital for vascular health, particularly under conditions of cerebral hypoperfusion. We also emphasize the complementary roles of Notch1 in ECs and Notch3 in mural cells, noting that downregulation of Notch signaling in both populations likely contributes to the breakdown of vascular integrity in CCH. The concurrent reduction of Notch signaling in these two critical cell types could compound the effects of CCH on the microvascular system, potentially accelerating the progression of cerebral small vessel disease. These insights suggest that future therapeutic strategies might benefit from targeting the preservation or restoration of Notch signaling in both ECs and mural cells, aiming to mitigate the vascular damage associated with chronic hypoperfusion.

Integrative analysis with published single‐cell RNA‐seq data revealed that upregulated DEGs were predominantly enriched in microglia, indicating enhanced activation of these immune cells. In contrast, downregulated DEGs were enriched in endothelial cells and pericytes, reflecting the adverse impact on these vascular components of the NVU. These distinct enrichment patterns underscore the cell‐type‐specific responses to CCH and highlight the complex interplay between different cellular constituents of the NVU. By integrating these findings, we offer a more detailed discussion of the transcriptional changes associated with BCAS and provide a clearer understanding of the complex interplay between endothelial and mural cell dysfunction in cerebral vascular diseases.

Vascular‐associated microglia (VAM) play a pivotal role in modulating the inflammatory milieu and influencing BBB integrity. They are increasingly recognized for their role in cerebrovascular diseases, acting as key players in the inflammatory response and contributing to neurovascular pathology seen in CCH [39, 40, 41]. Our study further underscores the role of microglial cells in modulating neurovascular responses to hypoperfusion, illuminating the intricate interactions between microglia and the vascular system. These results underscore the importance of microglial activity in the regulation and support of neurovascular integrity, particularly under conditions of cerebral hypoperfusion. The study provides new insights into the dynamic interplay between microglial cells and vascular components, revealing the critical contributions of microglia to the overall functionality and health of the NVU.

Overall, this study elucidates the dual impact of cerebral hypoperfusion on microglia‐associated neuroinflammation and Notch signaling. The results highlight the complex interplay between different cell types within the NVU and identify potential therapeutic targets for mitigating neuroinflammatory and vascular‐related neurodegenerative conditions. Future research should aim to further dissect the molecular mechanisms underlying these observations and explore therapeutic interventions that can modulate these pathways to preserve NVU integrity and function in the context of chronic hypoperfusion.

Despite the significant findings of this study, several limitations should be acknowledged. First, while the BCAS model effectively induces cerebral hypoperfusion, it may not fully replicate the complexity of human VCI and the variability seen in clinical conditions. Second, although our results highlight the downregulation of Notch1 in endothelial cells, we did not obtain data on Notch1 and Notch3 expression in mural cells or Notch4 in endothelial cells, which represents a limitation of this study. Third, while our results highlight the involvement of microglia‐associated neuroinflammation and Notch signaling, the precise molecular mechanisms and pathways remain incompletely understood. Further studies using additional models and techniques are necessary to elucidate these mechanisms. Additionally, therapeutic interventions targeting the identified pathways were not explored in this study, and future research should aim to validate potential treatments and their efficacy in mitigating neurovascular dysfunction and cognitive decline associated with chronic hypoperfusion.

## 5 Conclusion

In conclusion, this study provides critical insights into the molecular mechanisms underlying NVU alterations in chronic cerebral hypoperfusion. The findings demonstrate that CCH induces significant neurovascular changes, characterized by microglia‐associated neuroinflammation and downregulation of Notch signaling. These results highlight potential therapeutic targets for mitigating neuroinflammatory and vascular‐related neurodegenerative diseases, offering new avenues for research and clinical intervention in conditions associated with chronic hypoperfusion.

## Author Contributions

**Dewen Ru:** conceptualization, data curation, formal analysis, investigation, methodology, resources, validation, writing–original draft, writing–review and editing. **Zengyu Zhang:** conceptualization, data curation, formal analysis, investigation, methodology, resources, software, writing–original draft, writing–review and editing. **Meng Liu:** data curation, investigation, methodology, project administration, resources, writing–review and editing. **Xuhui Fan:** data curation, investigation, software, supervision, writing–review and editing. **Yuqi Wang:** data curation, funding acquisition, project administration, supervision, validation, writing–review and editing. **Yufeng Yan:** funding acquisition, supervision, validation, visualization, writing–original draft, writing–review and editing. **Ersong Wang:** conceptualization, data curation, funding acquisition, project administration, supervision; writing–review and editing.

## Ethics Statement

This study was performed in line with the principles of the Declaration of Helsinki. Approval was granted by the Ethics Committee of Shanghai Public Health Clinical Center (Approval number: 2023‐A040‐01).

## Conflicts of Interest

The authors declare no conflicts of interest.

## Supporting information

Supporting information.

Supporting information.

## Data Availability

The datasets provided in this study can be found in the online repository. The names of the repository/repositories and accession number(s) can be found below: https://www.ncbi.nlm.nih.gov/geo/query/acc.cgi?acc=GSE266214.
